# Tracking online topics over time: understanding dynamic hashtag communities

**DOI:** 10.1186/s40649-018-0058-6

**Published:** 2018-10-19

**Authors:** Philipp Lorenz-Spreen, Frederik Wolf, Jonas Braun, Gourab Ghoshal, Nataša Djurdjevac Conrad, Philipp Hövel

**Affiliations:** 1 Institute of Theoretical Physics, Technische Universität Berlin, Hardenbergstraße 36, 10623 Berlin, Germany; 2 Potsdam Institute for Climate Impact Research (PIK), Telegraphenberg A 31, 14473 Potsdam, Germany; 3 Department of Physics, Humboldt-Universität zu Berlin, Newtonstraße 15, 12489 Berlin, Germany; 4 Department of Physics and Astronomy, University of Rochester, Rochester, NY 14627 USA; 5 Zuse Institute Berlin (ZIB), Takustraße 7, 14195 Berlin, Germany; 6 School of Mathematical Sciences, University College Cork, Western Road, Cork, T12 XF62 Ireland

**Keywords:** Online media, Hashtags, Temporal community detection, Random walk, Memory matching, Topic dynamics, Ranking, Aging model, Bursts

## Abstract

**Background:**

Hashtags are widely used for communication in online media. As a condensed version of information, they characterize topics and discussions. For their analysis, we apply methods from network science and propose novel tools for tracing their dynamics in time-dependent data. The observations are characterized by bursty behaviors in the increases and decreases of hashtag usage. These features can be reproduced with a novel model of dynamic rankings.

**Hashtag communities in time:**

We build temporal and weighted co-occurrence networks from hashtags. On static snapshots, we infer the community structure using customized methods. On temporal networks, we solve the bipartite matching problem of detected communities at subsequent timesteps by taking into account higher-order memory. This results in a matching protocol that is robust toward temporal fluctuations and instabilities of the static community detection. The proposed methodology is broadly applicable and its outcomes reveal the temporal behavior of online topics.

**Modeling topic-dynamics:**

We consider the size of the communities in time as a proxy for online popularity dynamics. We find that the distributions of gains and losses, as well as the interevent times are fat-tailed indicating occasional, but large and sudden changes in the usage of hashtags. Inspired by typical website designs, we propose a stochastic model that incorporates a ranking with respect to a time-dependent prestige score. This causes occasional cascades of rank shift events and reproduces the observations with good agreement. This offers an explanation for the observed dynamics, based on characteristic elements of online media.

## Background

Networks of complex systems represent functional or contextual relations that show globally and locally heterogeneous substructures. One important feature is the densely interconnected groups of nodes, which are called communities. Their organizational arrangements can have various characteristics such as overlapping, fuzziness or hierarchical structure and require diverse detection algorithms [1–4].

Time-resolved data of online content has become increasingly available and is of great importance for understanding the dynamics of content, including the emergence and lifetime of topics or trends. The development of methods, which capture these temporal communities is a subject of current research [5–7]. Moving from a static to a temporal picture requires tracking the communities in time. This naturally raises the question of a temporal matching of communities resulting from static snapshots [8–12]. By incorporating higher orders of memory [13] in a method proposed in [14], long-term developments can be tracked reliably.

The temporal aspect of this approach is independent from the choice of static community detection algorithm and provides a free parameter to define the timescale of a thread in order to meaningfully define a topic. The proposed method can track trajectories of content on various timescales that can occur for instance in the highly dynamical world of online media. Especially long-term developments can be followed well by canceling out noise and by memorizing topics even with interruptions due to daily or weekly periodicities.

Previously in [14], we introduced a random-walk approach for hashtag community detection and a subsequent memory-based matching scheme on temporal networks. We had demonstrated that the lifetime of small communities can be increased with this approach. This paper serves as a substantial extension of the said conference paper. This paper provides a more detailed motivation of the approach, a detailed description of the matching procedure, including a discussion of different memory kernels and in a completely new part, focuses on analyzing the empirical dynamics of the groups that we can trace over time. Furthermore, we will elaborate on a mechanistic model to reproduce and understand their features. The resulting trajectories of hashtag groups allow us to analyze the way these groups grow and shrink. If a topic is new and widely discussed, people start inventing hashtags for its description. They are combined with established and popular hashtags, for the posts to appear in many queries and reach many users. This leads to an imitation or preferential attachment behavior as often observed in other social settings [15]. Simultaneously the total volume of hashtags that are posted within a topic decreases after some time. Other topics come up and the discussion will eventually switch to new subjects and their corresponding hashtags, leading to a cycle similar to the news media [16].

This behavior leads to a fat-tailed distribution of increases of group sizes in agreement with observations in other systems [17, 18]. It has been described based on a ranking model for network growth [19] via exogenous and random shifts. We, however, observe also bursty behavior in the decreases of the communities. To account for this, we extend the existing models by a recency ranking and gain a deeper understanding of the complex dynamics of the ever-changing usage of hashtags.

## Hashtag networks

**Fig. 1:**
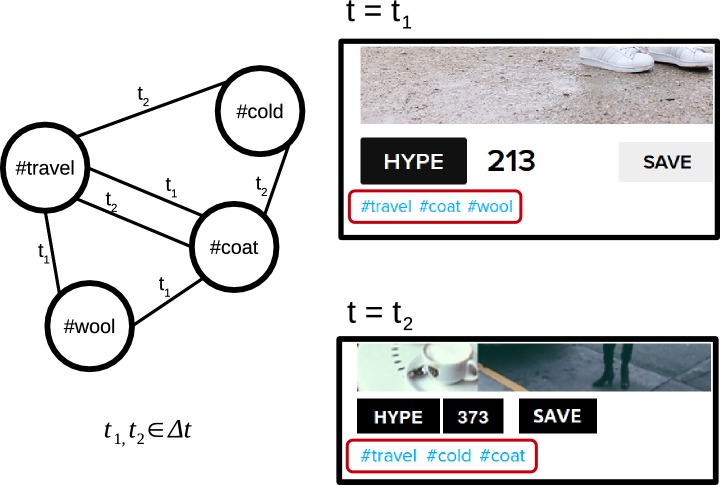
Scheme for the construction of co-occurrence networks of hashtags: every time two hashtags are used within the same posting, and an edge with the timestamp of that post is drawn. Aggregating the edges over a time-window results in an undirected and weighted snapshot network. On the right, screenshots from https://lookbook.nu

In order to analyze groups of related content with methods from network science, we build co-occurrence networks from empirical datasets. In this work, we will focus on hashtags from the fashion platform https://lookbook.nu, where users can post pictures of outfits to their followers and describe them with hashtags. The dataset was acquired in April–May 2017. An HTML scraper was used to extract information from the public webpages of lookbook.nu via HTTP. Starting at a random user, 22,748 users were crawled along the follower-connections in order to focus on popular accounts. These users produced 1,158,340 posts within the observation time, which contained 81,409 unique hashtags in total. Nodes are labeled with corresponding hashtags and edges are realized, whenever two hashtags occur in the same posting, similar to network constructions that have been used to analyze social tagging systems [20]. These edges are undirected and timestamped, ranging through the complete year 2015. Aggregating them within a time interval $\Delta t$ results in snapshots of the temporal network. To account for multiple co-occurrences within $\Delta t,$ we introduce corresponding edge weights. The snapshots can also be represented as weighted adjacency matrices $A_t,$ with zero or positive integer elements [21]. Figure 1 illustrates this procedure schematically. The aggregated network over the complete dataset has a total size of 81,409 nodes, connected by 1,358,241 edges. To analyze the temporal evolution of these connections, we used smaller time intervals. In this work, we choose an aggregation window of 1 week ($\Delta t = 7$ days) in order to avoid structural changes due to patterns within a week. As a result, we obtain 52 snapshot networks for 2015. Standard measures of these networks, averaged over all snapshots, are the mean degree $\langle k\rangle = 6.2,$ the diameter $D = 5.03,$ and the mean path length $\langle l\rangle = 3.4$ as well as the global clustering coefficient $C = 0.62.$ These values are comparable to word co-occurrence networks [22] and remain stable over time.

### Community structure

**Fig. 2:**
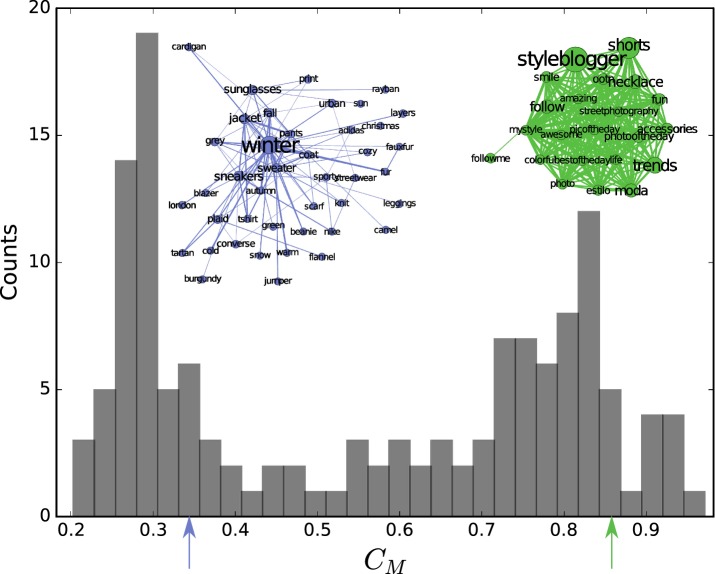
Two types of groups: histogram of the global clustering coefficient of each community found with modularity maximization. The inset shows two examples of substructures that we observe in the low- and high-value areas. A hierarchically ordered structure shown in purple and a densely connected group in green. These network visualizations and those in Fig. 5 are generated using gephi [38]

Since hashtags can be used in different contexts by diverse communities of people, we suspect a formation of strong substructures in such networks. The modularity value is relatively high ($Q > 0.5$) for all snapshots, suggesting pronounced subgraphs [23]. Modularity maximization gives a good possibility to get a first impression of these structures [24]. Figure 2 shows the distribution of the global clustering coefficients $C_M$ of each individual module *M* from 52 snapshots. The bimodal character suggests that mainly two structural types can be found. We hypothesize that this corresponds to different ways of using hashtags: A descriptive usage of hashtags as keywords results in structures with lower clustering coefficient (example: Fig. 2, purple inset), while the usage of high numbers of buzzword hashtags in each post shape strongly clustered groups (example: Fig. 2, green inset).

**Fig. 3:**
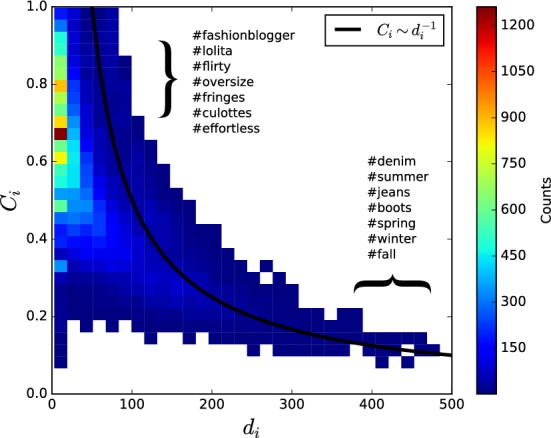
Hierarchical structured and unspecific hashtags: the two-dimensional distribution of value pairs $(C_i,\;d_i),$ clustering coefficient and degree of node *i*, respectively. The most frequently used hashtags, which fall into the region within the curly brackets, are listed (upper left: $0.6< C_i < 0.8,$ lower right: $300< d_i < 500$)

This picture is supported by investigating the relation of degree and local clustering coefficient. Figure 3 shows the distribution of combinations $(d_i,\;C_i)$ of degree $d_i$ and clustering coefficient $C_i$ for each node *i* across all snapshots. A majority of the networks follow the relation: $C_i \sim d_i^{-1}$ as described in [25] for hierarchical networks. Low clustering coefficients define top level hashtags for broader topics (e.g., ’#summer,’ ’#denim’). The upper part of the distribution contains very specific hashtags, as expected in hierarchical networks, but also less meaningful buzzwords (e.g., ’#fashionblogger,’ ’#effortless’).

Considering their topological position in the network (Fig. 5a), we observe that nodes with high clustering coefficient either lie in the periphery of hubs (Fig. 5b), or they shape strongly intraconnected groups. This leads to the picture of networks that consist of several hierarchically structured subgroups that share nodes in their periphery, which have large clustering coefficients. Figure 5c shows how modularity maximization can be misled in such networks by combining hubs that do not belong together. To separate the topics from each other and possibly filter out the unspecific groups between them, we incorporated our understanding of the data in a customized community detection method.

### Finding hashtag communities

Our approach is to adapt a time-continuous random-walk (RW) clustering method developed in [26, 27]. This method is based on exploiting dynamical properties of the RW to find communities such that they correspond to the metastable sets of the process, i.e., structures where the RW is stuck for very long time periods. To achieve this, we define a new type of a time-continuous random walk such that hashtag communities represent its metastable sets. The dynamics of this new process are given by the following rate matrix:

1 $$\begin{aligned} L_\phi(i,\;j) = {\left\{ \begin{array}{ll} -\frac{1}{e^{\phi (1-C_i)}}, & \quad i=j\\ \frac{A_{i,\;j}}{d_i e^{\phi (1-C_i)}}, & \quad i\ne j,\quad A_{i,\;j} > 0 \\ 0, & \quad \text {else}, \end{array}\right. } \end{aligned}$$

where *A* is the weighted adjacency matrix, $d_i$ is the degree, and $C_i$ is the clustering coefficient of a node *i*. Parameter $\phi >0$ is a constant that is used to regulate the general importance of $C_i$ depending on the given data. Transition rates from a node *i* to a node *j* are given by the off-diagonal elements of $L_\phi.$ Diagonal elements indicate the metastability of the process within hashtag communities, since the expected waiting time in every node *i* is given by $\frac{1}{\Vert L_\phi (i,i)\Vert } = e^{\phi (1-C_i)}.$ Therefore, a process stays longer on average in nodes with smaller values of the clustering coefficient. By taking into account both local measures and topological information, we achieve two things: hubs are naturally often visited, while the densely connected groups between them are not attractive for the random walker and it passes through them quickly.

Now, we can find the hashtag communities $M_1,\ldots , M_m$ as metastable sets of the RW process given by Eq. (1). For this, we use the Markov state modeling approach [27], as it provides a way to find fuzzy communities and filter out unspecific hashtags. In particular, we obtain clustering into communities $M_1,\ldots , M_m$ and additionally a transition region $T = {V\setminus}\left(\bigcup _{l=1}^m M_l\right),$ consisting of the remaining nodes from the set of all nodes *V*, which are not uniquely assigned to exactly one of the communities. A transition region can act as a filter for very unspecific hashtags by accounting for the typical fuzzy character of communities in tag co-occurrence networks, avoiding overlapping areas [28, 29]. For nodes in *T,* we can calculate the affiliation probability to each $M_1,\ldots , M_m$ by solving sparse, symmetric, and positive definite linear systems [26, 30].

**Fig. 4:**
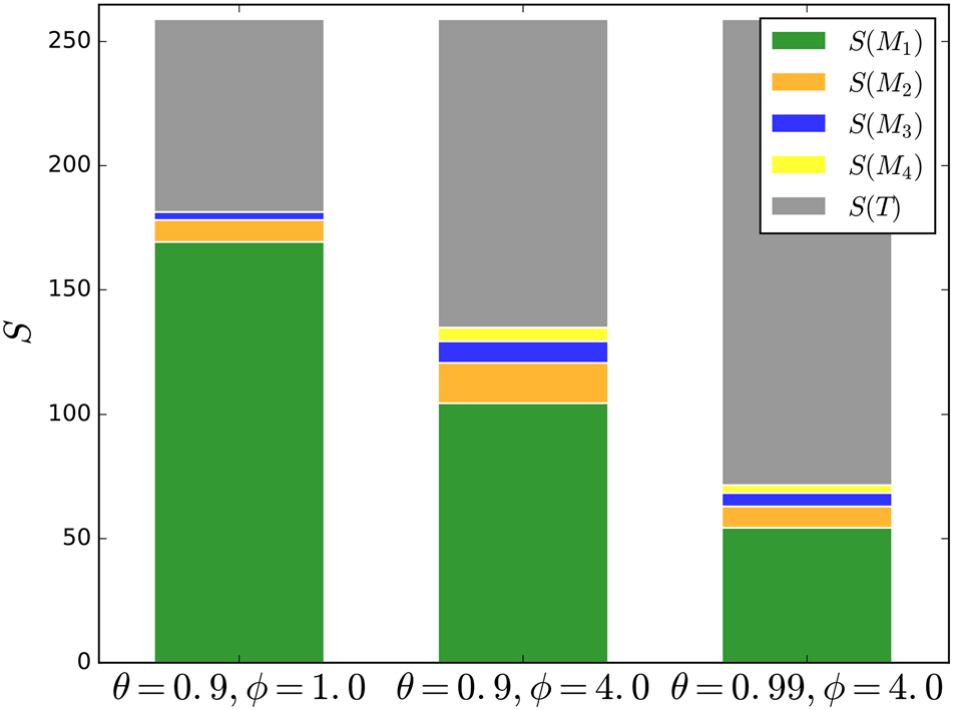
The roles of $\phi$ and $\theta{:}$ three examples of the same simple network but with different module compositions resulting from different combinations of the parameters $\phi$ and $\theta$ from the RW method. The module sizes $S(M_i)$ and the size of the transition region *S*(*T*) as the number of nodes they contain are shown

Details of this approach are described in [26, 30], and in the following, we briefly highlight the effects of two parameters that control the main components of this method: $\phi$ controls the repulsive force of high local clustering coefficients $C_i$ , and $\theta$ sets the lower threshold of the affiliation probability. Figure 4 shows three examples of community composition in the same simple network but, for different parameter combinations. For low values of $\phi,$ the rate matrix (see Eq. (1)) allows transitions through regions of high clustering coefficients. Walks between high-degree nodes become more favorable, and the transition region *T* between them becomes small. Increasing $\phi$ separates the hubs and leads to the highest diversity in the modules and their sizes, where $\theta =0.9$ and $\phi =4.0$ (see Fig. 4). A higher value for $\theta$ increases the size of the transition region even further, shrinking the smaller communities, leading to less homogeneous sizes again.

**Fig. 5:**
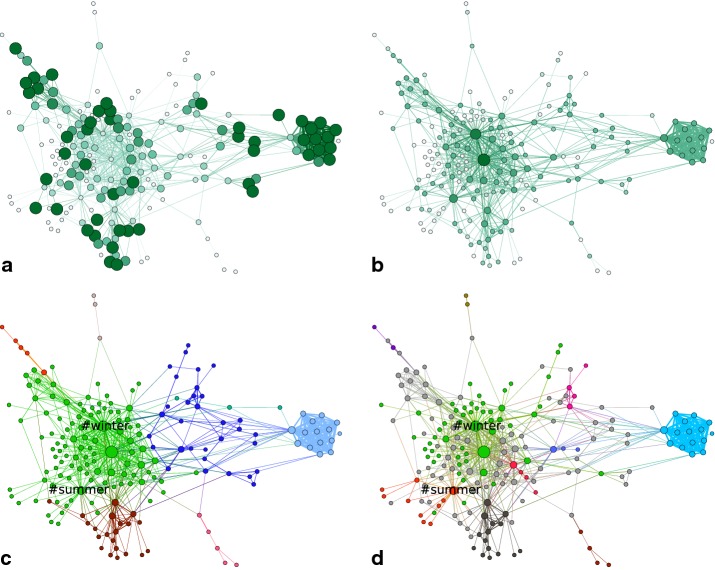
Values of two local network measures and their effects on community detection: **a** the local clustering coefficients in one network snapshot and **b** the degrees of the nodes. The darker the color and the larger the radius of a node, the higher the value of a measure. The two pictures show the same network, with fixed node positions. **c** The same network, with node colors corresponding to the modules that were obtained by modularity maximization (edge colors follow the colors of the attached nodes for better visualization) and **d** obtained by our customized RW method, resulting in a fuzzy clustering with the transition region *T* in gray

Comparing modularity maximization for weighted graphs to our method in Fig. 5c, d leads to a similar insight. The lack of a transition region destabilizes modularity maximization and leads to merging groups of different topics that should be separated in our analysis. Depicted is an example, where the hashtags #summer and #winter get assigned to the same group by modularity maximization, while they are separated by the transition region (gray) with our method.

The described method has two advantages: (1) It can be customized to our needs for the characteristic underlying network structure, by modifying the RW process. (2) It can detect fuzzy communities, which is important property of the hashtag groups under investigation and possibly more general for word groups. It is outside the scope of this work to compare our method to other community detection methods, in part because our method is designed to infer fundamentally different topological structures. The remaining parts of this work are independent of the community detection on the static snapshots, allowing for a customized solution as the one presented above.

## Dynamics of communities

**Fig. 6:**
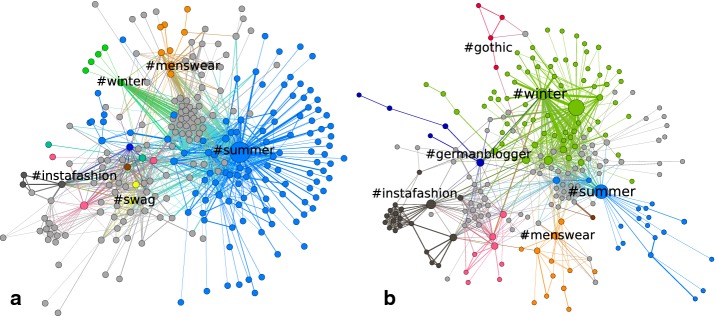
Two representative snapshots, with clustering: **a** the resulting communities on a snapshot from August, **b** the results for a week in December. Colors correspond to different communities $M_i$ and gray nodes form the transition region *T*

The fashion world underlies strong seasonal and trend-driven changes, which lead to alterations in the hashtag landscape. In Fig. 6, two snapshots,  a week in August and a week in December, are shown. It can be observed that the community structure varies largely between the two seasons. Understanding the dynamics of these developments requires a method to quantitatively capture the communities over time. We propose a meta-algorithm that solves the bipartite matching problem, which arises from connecting previously obtained partitions of every snapshot network. It is important to note that this method is independent from the choice of the algorithm used for the static community detection on the individual snapshots. Generally, the class of matching-based methods for temporal community detection [8–11, 14] offers a big advantage, by allowing us to choose a static detection method for the specific data structure and question.

### Matching problem

To measure properties like stability, and the rise and descent or the lifetime of communities, we track their history through the snapshot networks. In contrast to an event-based approach [9], our goal is to find long-term developments and re-identify forgotten trends rather than observe behavioral patterns of various events. Our first assumption is that the vocabulary used to talk about a topic stays similar from one day to the other. This directly suggests maximizing the sum of pairwise similarity measures for adjacent timesteps. For example, one can compute the overlap of hashtags of two communities, *A* and *B*, from the snapshots at $t-1$ and *t*, respectively, by considering their Jaccard index:

2 $$\begin{aligned} J\left(A_{t-1}, B_t\right) = \frac{\vert A_{t-1} \cap B_t \vert }{\vert A_{t-1} \cup B_t \vert }. \end{aligned}$$

**Fig. 7:**
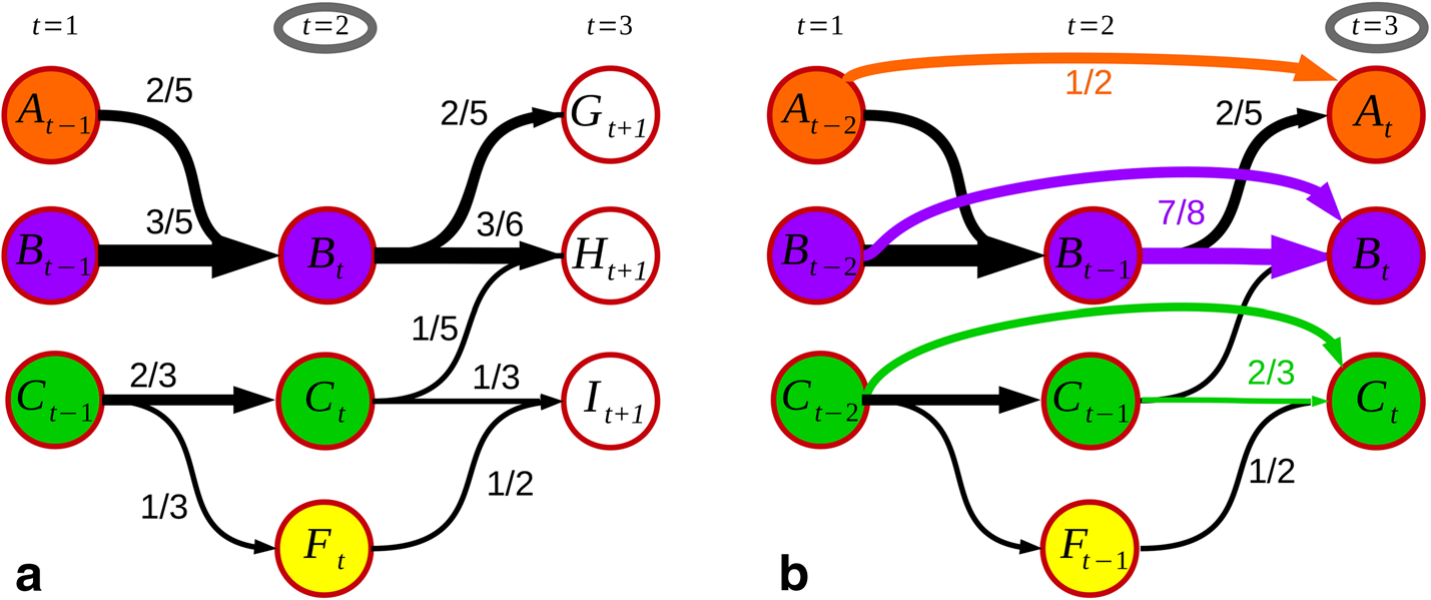
The matching process: **a** pairwise calculated Jaccard indexes and the resulting coloring in step $t=2.$ **b** The memory weights *M* for some groups and their effects on the matching in step $t=3$

Using the above, we construct a weighted bipartite graph with hashtag communities as vertices and weighted edges with the Jaccard index as schematically drawn in Fig. 7a. Jaccard indexes below a threshold $J_t = 0.1$ are not considered in that construction, a lower bound that can be varied according to the desired minimal overlap. In order to track the groups over time, we face a matching or coloring problem on that graph, which can be solved by the Hungarian method in polynomial time [31].

In this first example, the matching is simple, namely, the one that results in the maximal sum of Jaccard indexes $J_{\text{max}} = 3/5 + 2/3 = 1.27.$ All groups, for which a matching was found, are then renamed to be consistent with the labeling from the previous timestep (Fig. 7a). Names of communities which could not be matched, like $F_t,$ are kept. This renaming procedure gives the possibility to track the development of a community over time and to measure its lifetime or the changes of its size.

### Memory weights

The discrete nature of community detection makes it generally unstable toward variations in the network topology. Communities split (cf. *F* in Fig. 7a) or merge (cf. *A*) due to small temporal topological changes, but can reunite or separate after only one step. This can lead to unwanted effect, as in the third timestep $t=3$ from our example, where a pairwise Jaccard index finds no match for *G* and it is understood as a new development. Similarly, *I* is identified as *F*, while there will not be any match for *C* and its development would stop. However, these events are just temporal fluctuations and should not deter the continuity of groups *A* and *C*. To overcome this algorithmic deficiency, we expand the task to a multistep matching. We recursively consider possible matchings from snapshots further in the past within a time window of length *n*. As similarity measure, we sum up the Jaccard indexes over the *n* preceding steps, weighted by the inverse temporal distance to compute memory-dependent weights *W*:

3 $$\begin{aligned} W(\lbrace A_{t-n}, ..., A_{t-1} \rbrace , B_t) = \sum _{t^{\prime}=1}^n \frac{1}{t'} \frac{\vert A_{t-t^{\prime}} \cap B_t \vert }{\vert A_{t-t^{\prime}} \cup B_t \vert }. \end{aligned}$$

This proposed protocol of calculating the weights incorporates the ideas of considering timesteps further in the past [11] as well as a finite length of influence [10]. This is motivated by the assumption that a topic can be followed over time as long as a fraction of its members stay the same for a finite timespan even if members change in the long run. In Fig. 7b, two possible scenarios are illustrated. The group *A* has disappeared but can be rediscovered by the value $W\left(\lbrace A_{t-2},\;A_{t-1}\rbrace,\;H_t\right) = 1/2,$ which is higher than $W \left(\lbrace B_{t-2},\;B_{t-1}\rbrace,\;H_t\right) = 2/5.$ The other scenario is the small group *F* that split off *C* but merges back afterwards. The memory accounts for that by a high overlap $W\left(\lbrace C_{t-2},\;C_{t-1}\rbrace,\;J_t\right) = 2/3$ and results in keeping the label *C*. The choice of the window size *n* depends on the data, but also on the natural timescales of the dynamical processes that are of interest. If it, for example, is not desirable to relabel a group when it undergoes weekly periodicities, one should choose the window to be longer than a week. Following the goal to capture developments that have timescales of months, we use $n=4$ weeks to explore the seasonal trends in fashion.

Alternatively, we can sum over all available timesteps using an exponential memory kernel with decay rate *r* to compute *W*:

4 $$\begin{aligned} W(\lbrace A_{t-n}, ..., A_{t-1} \rbrace , B_t) = \sum _{t^{\prime}=1}^t e^{-t'\cdot r} \frac{\vert A_{t-t^{\prime}} \cap B_t \vert }{\vert A_{t-t^{\prime}} \cup B_t \vert }. \end{aligned}$$

The above version of the memory weights has the advantage that we can extract a proxy for the value of *r* from the data. Calculating the average relative overlap of all hashtags *H* between adjacent snapshots $\left\langle \frac{H_t \cap H_{t+1}}{H_t \cup H_{t+1}} \right\rangle _t \approx 0.9$ naturally suggests a choice $r=0.1.$

### Testing stabilization

**Fig. 8:**
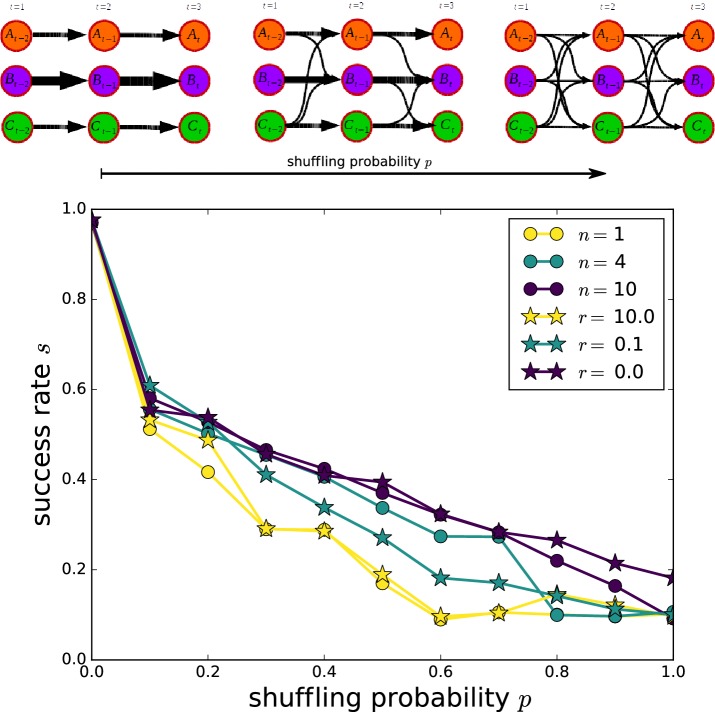
Testing stabilization: illustration of a benchmark test to quantify the ability of stabilization in noisy data for different parameters. The resulting success rates *s* are shown for different scenarios with two memory kernels ($1/t^{\prime}$ and $e^{-r \cdot t^{\prime}}$) and range from the limit of no memory ($n=1$ and $r=10.0$) to the case of infinite memory ($n=10$ and $r=0$)

The advantage of our method based on Eqs. (3) and (4) to find a matching in noisy data can be quantified by a constructed test case. To this end, we start with a static partitioning and generate uncorrelated randomized copies of it by swapping members between the communities with a fixed probability *p*. The obtained randomized snapshots can be assembled one after the other to construct a noisy time series with a stable underlying community structure (Fig. 8). One can then run the matching procedure on this artificial timeseries and quantitate how often the matching algorithm found the underlying (known) groups in the noisy data by the relative success rate *s*. The resulting values for different shuffling probabilities *p*, depending on memory lengths *n* and decay rates *r*, are compared in Fig. 8. The case of $n=1$ corresponds to a usual Jaccard index-based matching. By means of only a few steps of memory, the accuracy can be increased significantly, especially for relatively low shuffling probabilities. For strongly randomized matchings, only high memory values can still find the underlying structure. The empirically measured decay rate $r=0.1$ as well as the finite window size of $n=4,$ which we use throughout this work, achieve both good success scores, especially in the more realistic regime of low shuffling probabilities.

### Empirical results

**Fig. 9:**
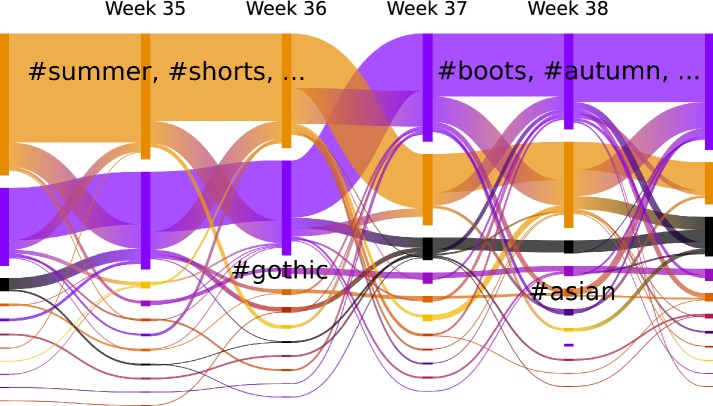
Alluvial diagram during the change of seasons: 6 weeks at the end of August of our data visualized. The number of hashtags in each group and transition is encoded in the thickness of the drawing, while the groups are ordered by their size

An insightful visualization of such complex datasets with temporal community structure was proposed in [32] under the name ’alluvial diagrams.’ Figure 9 depicts one example for such a diagram, where the communities are drawn for each snapshot and the hashtags transitions between them, encoded in the thickness of the bands. The ’#summer’ community loses many members and the ’#autumn’ group becomes the biggest one in the 1st week of September. Our interactive online tool can be used further to explore the results (www.tu-berlin.de?lorenz). In Fig. 9, the community sizes vary largely, showing many small but stable groups such as the exemplarily labeled ‘#gothic’ and ‘#asian’ developments. These small groups suffer the most from fluctuations. Besides these results, an implementation of the matching method is available at: https://github.com/philipplorenz/memory_community_matching.

## Modeling online dynamics

**Fig. 10:**
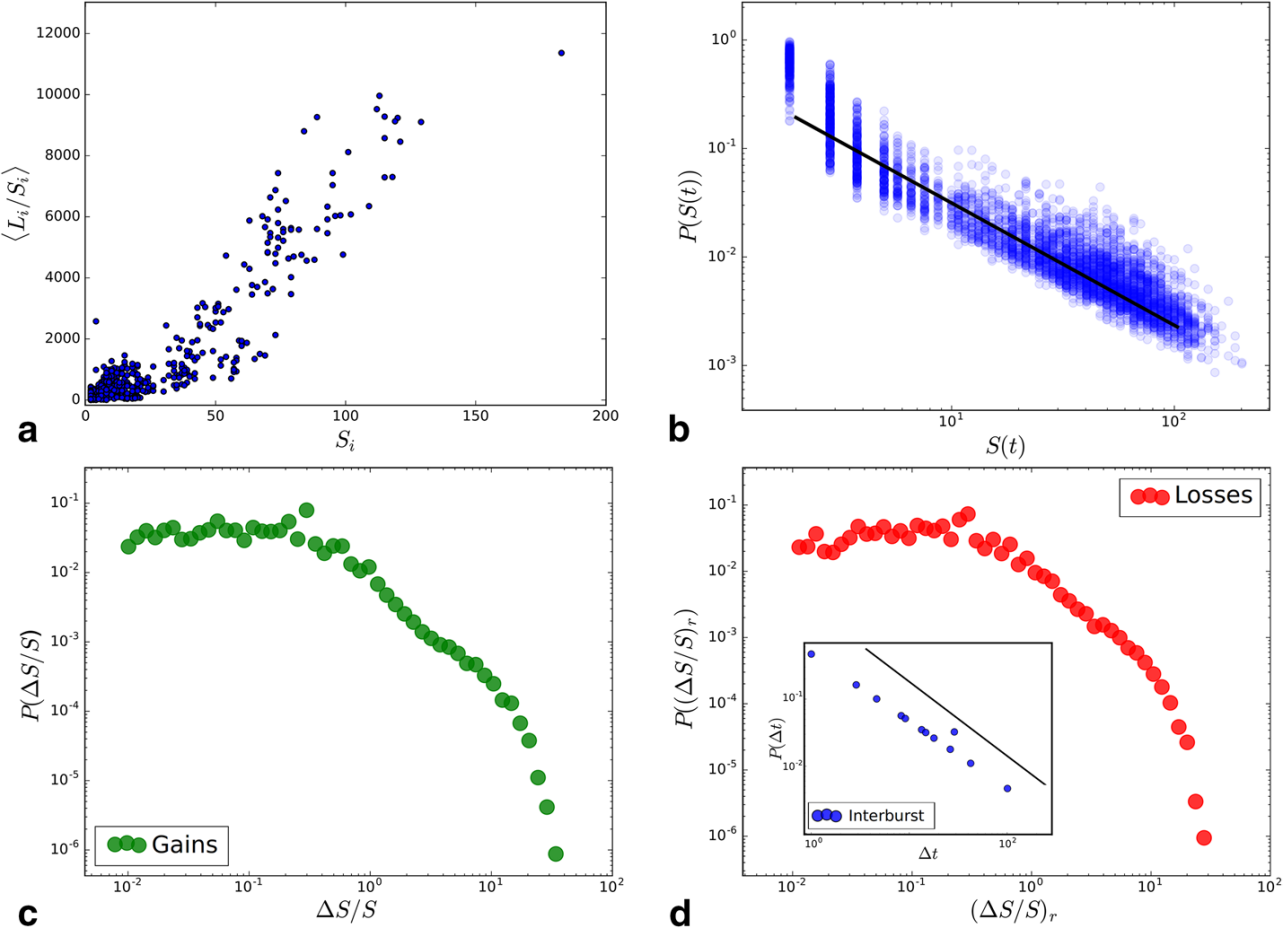
Statistics of various observables: **a** the correlation between the average likes $\langle L_i/S_i \rangle$ per hashtag of community *i* and its size $S_i.$ **b** The distribution of (community-)sizes in each snapshot *S*(*t*), plotted on top of each other and as a guide to the eye, a fitted power law (exponent: $1.27\pm 0.03,$ KS-statistic: 0.2, *p* value: 0.08). **c** The distribution of relative gains $\Delta S / S = (S(t)-S(t-1))/S(t-1)$ (green) in size and **d** relative losses $(\Delta S / S)_r = (S(t-1)-S(t))/S(t)$ (red). The Inset shows the distribution of interburst times $\Delta t$ between events of $\Delta S / S > 10$ (blue) and a fitted power law (exponent: $1.1\pm 0.02,$ KS-statistic: 0.3, *p* value 0.05)

The proposed methodology enables us to observe highly dynamic developments of hashtag groups rising and falling in their size (see Fig. 9). By a mathematical model, we aim to understand the main driving forces that cause people to post new combinations of hashtags and to drop a topic again. To this end, we focus on the development of the sizes $S_i(t)$ of community *i* at time *t*. Our dataset includes the likes *L* that are placed on the postings, and we could observe a strong correlation of average likes per hashtag $\langle L_i/S_i \rangle,$ a community receives and its size $S_i,$ shown in Fig. 10a (Pearson correlation $\rho =0.925$). In the following, we treat its size as a proxy for its popularity, and the quantity, which is simulated by the proposed model, can be understood as conceptual score for popularity.

Figure 10b shows the distributions *P*(*S*(*t*)) of total sizes in each week. They are plotted on top of each other to illustrate their stability and shape. We used the maximum likelihood method from [35] to estimate the exponent and the standard error of the indicated power law. This shape fits to the general picture of many distributions that are related to popularity measures.

**Fig. 11:**
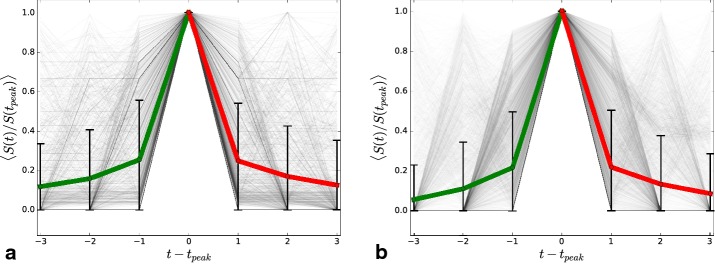
Observed peaks and their simulation: **a** All trajectories $S_i(t)$ leading to and from the global maximum $S_i(t_{\text{peak}}),$ relative to that $S_i(t)/S_i(t_{\text{peak}}).$ Their mean s are plotted in green (increase) and red (decrease) with the corresponding standard deviation at each point. **b** Exactly the same plot for the resulting trajectories obtained from a Monte-Carlo simulation of Eq. (9) with parameters: $\alpha = 3.0,$ $m=500,$ and $N = 100$

In the following discussion, our main focus is on the dynamics of these values, which exhibit very diverse temporal evolution. The trajectories before and after a maximum are shown in Fig. 11a. Their mean values (green and red) show very symmetric behavior of gaining and losing members, while the large standard deviations confirm their broadly distributed *S*. To quantitate this further, we consider the distributions of the logarithmic derivative $\Delta S / S = (S(t)-S(t-1))/S(t-1)$ (green), as was previously done in [17, 34], which describes the relative gains. In addition, in this work, we describe the relative losses $(\Delta S / S)_r = (S(t-1)-S(t))/S(t)$ (red) from 1 week to the other. Both their distributions are plotted in Fig. 10c, d.

We observe in agreement with Fig. 11a that both distributions are very similar and exhibit a fat tail. A similar behavior has been demonstrated for relative gains in Wikipedia traffic [17], Youtube views [18], hashtag usage on Twitter [33], and citation counts [34]. These fat-tailed distributions of relative changes seem to be a characteristic feature of online popularity dynamics.

Pure rich-get-richer mechanisms, where $\Delta S \sim S$ cannot reproduce these broad distributions; however, their shapes suggest self-enhancing process in both directions (gain and loss). Exogenous events can be responsible for small items to rise up quickly as modeled in [17], but also aging of the top items [34] can lead to rising newcomers. The distribution of interevent times (event: $\Delta S / S > 10$), as shown in the inset of Fig. 10d, is following a power law, where we also used the method from [35] to estimate the exponent. This is an indicator of cascades of events, which we believe are caused by competition among pieces of content [36], e.g., by the downfall of a popular item, leaving room for others.

In the following, we propose a class of models that is able to explain these observations by an interplay of ephemeral popularity and ranking mechanisms.

### Ranking

https://lookbook.nu and many other websites show posts in a longitudinal order, naturally imposing a hierarchy. It is a natural tendency to sort items according to their relative attribute. This is also what most websites do, their algorithms are usually called ’trending,’ ’hot,’ ’popular,’ or ’new.’ To account for this, the proposed model incorporates a ranking as previously described in [17, 19, 33]. We adapted this basic idea by ranking community *i* according to a prestige score $\lambda _i(t),$ which depends on time. To order these scores relative to each other, we formulate a general ranking function $r(\lambda _i(t), \lbrace \lambda _1(t), ... ,\lambda _N(t) \rbrace )$ as the sum of Heaviside functions:

5 $$\begin{aligned} r(\lambda _i(t), \lbrace \lambda _1(t), ..., \lambda _N(t) \rbrace )&:= \sum _{k=1}^N \Theta ( \lambda _k(t) - \lambda _i(t) ) + 1 \end{aligned}$$

6 $$\begin{aligned} \text {with} \quad \Theta (x)& = {\left\{ \begin{array}{ll} 0, \quad \text {if }x<0\\ 1, \quad \text {if }x \ge 0. \end{array}\right. } \end{aligned}$$

For simplicity of the notation, we neglect its dependence on all other states and consider it as implicitly present $r(\lambda _i(t), \lbrace \lambda _1(t), ..., \lambda _N(t) \rbrace ) \equiv r(\lambda _i(t)).$ The function results in a small integer $r(\lambda _i(t))$ if an item *i* has a high score $\lambda _i$ relative to the others. By that coupling, the ranking implies a competition between the topics *i*, due to a limited capacity of the users and websites.

In each discrete timestep, the score $S_i(t+1)$ a topic *i* receives is distributed according to the ranks, which result from the last timestep. Following are the attachment probabilities:

7 $$\begin{aligned} P(r(\lambda _i(t)) = \frac{r( \lambda _i (t))^{-\alpha }}{\sum _{j=1}^N r(\lambda _j (t))^{-\alpha }}. \end{aligned}$$

The attachment probability decreases with $P(r(\lambda _i(t)) \sim r^{-\alpha }$ modeling the decay of user attention as they scroll further down the feed and is consistent with the observed decay of sizes (Fig. 10b). Consistent with the momentary character of the empirical popularity measure $S_i(t),$ we model the users to newly distribute *m* scores (e.g., likes) along this feed of posts at every time step. This can be expressed in the following update rule:

8 $$\begin{aligned} S_i(t+1) = \sum _{h=1}^m \Theta (P(r(\lambda _i(t)) - \xi )), \end{aligned}$$

where $\xi \in \left[ 0, 1\right]$ is a uniformly distributed random variable and $\Theta (x)$ as defined in Eq. (5). If an item has been ranked down, resulting in a lower attachment probability ($P(r(\lambda _i(t)) < P(r(\lambda _i(t-1))$), this update rule can cause decreasing scores $S_i(t+1) < S_i(t)$ (losses).

### Aging model

In the ranking of community sizes, constant turnovers can be observed in Fig. 9 as well as negative slopes $\Delta S < 0$ (Figs. 10d and 11a) that refer to shrinking processes. To account for this, it is necessary to introduce, besides the rich-get-richer mechanism, an age-dependent decay of the prestige score. The age of a node has been considered in [37] by the assumption that old nodes might not attract as many new links as young ones and slow down in growth. In our case, we assume that hashtags/topics, especially describing pop-culture and news, have to be up to date. If they lack recency, they are mentioned by less users with time [16]. To this end, we rank the topics by a combined score of attractiveness, namely, the differences of their sizes and their ages $\lambda _i(t) = S_i(t) - a(t-t_i),$ where *t* is the current time, $t_i$ the time of introduction. The aging factor *a* weights the influence that the age has on the ranking. This leads to the following attachment probabilities in the aging model:

9 $$\begin{aligned} P(r(\lambda _i(t))) = \frac{r(S_i(t) - a(t-t_i))^{-\alpha }}{\sum _{j=1}^N r(S_j(t) - a(t-t_j))^{-\alpha }}, i=1,...,N. \end{aligned}$$

Alternative choices of prestige scores $\lambda _i(t)$ are possible. The general dynamic behavior occurs whenever this score eventually decays with time. An interesting option for future research, e.g., is the rate of change $\lambda _i(t) = \Delta S_i(t).$

### Numerical results

**Fig. 12:**
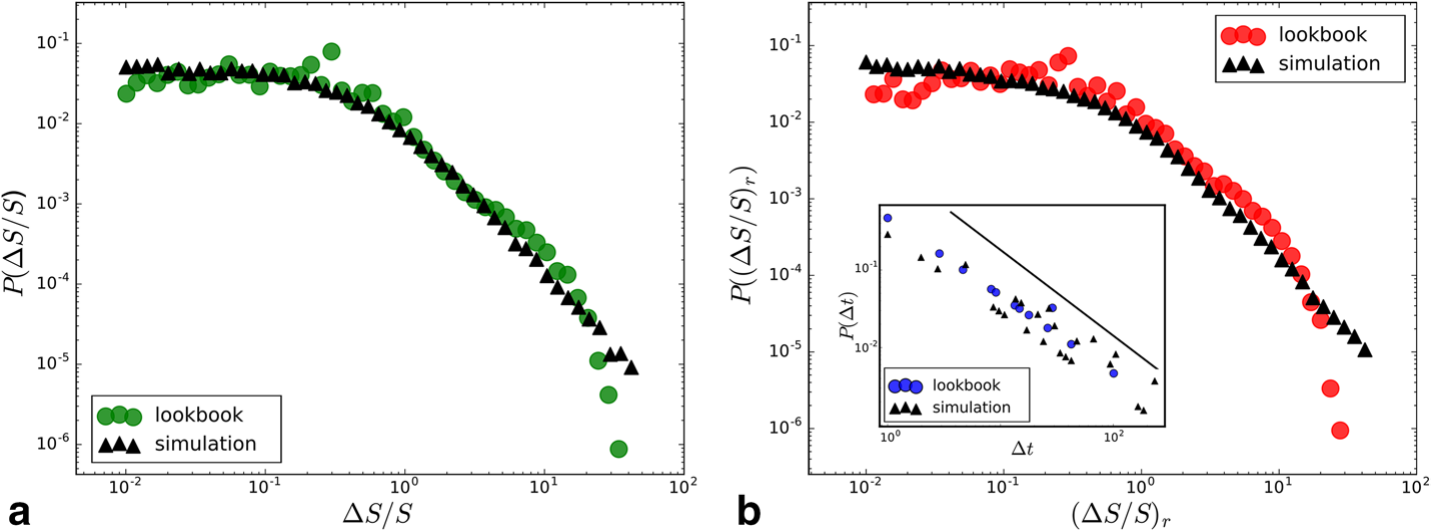
Comparison of empirical and simulated distributions **a** distributions of gains $\Delta S / S = (S(t)-S(t-1))/S(t-1),$ empirical (green) and simulated (black). **b** Analogous to the distribution for losses $(\Delta S / S)_r = (S(t-1)-S(t))/S(t)$ (red). The Inset shows the distribution of interburst times $\Delta t$ between events of $\Delta S / S > 10$ from the data (blue, fitted exponent: $1.1\pm 0.02$) and the simulation (black)

Equation (8) can be implemented and simulated. For simplicity, we keep the total number of topics $N={\text{const}}.$ in this work by adding a new topic each timestep, while removing the smallest one. Adding new hashtags to the system with $S_i(t=t_i) = 0$ accounts for exogenous events. The resulting trajectories and their mean s are shown in Fig. 11b, with good agreement to the empirical observations. The simulation of our model with $\alpha = 3.0,$ $m=500,$ $a=1.0,$ and $N = 100,$ reproduces both distributions shown in Fig. 12a, b very well with a Kolmogorov Smirnov distance of 0.05 (*P* value $<0.001$) for the gains and 0.06 (*p* value $<0.001$) for the losses. The distribution is broad due to cascades of rank shifts. The lack of regularity and the burstiness of these jumps become clear in the power-law distribution of interburst times between events of $\Delta S / S > 10$ as shown in the inset of Fig. 12b.

### Staying on top

In the simulated dynamics, it seems that higher ranks are held longer while the frequency of rank-shift events increases between lower ranks. In order to quantitate this, we formulate the condition that an item *i* in rank *r* loses its position to item *k* from the rank below $r+1{:}$

10 $$\begin{aligned} S_i(t) - a(t-t_i) < S_k(t) - a(t-t_k). \end{aligned}$$

As a lower bound for the time to stay in a rank, we assume that the ranks are reached adiabatically fast compared to the time they stay there. Then, we approximate the competitor *k* from the lower rank $r+1$ to be very young $t_k \approx t$ and the score on rank *r* to be on average $\langle S(r) \rangle = m \cdot P(r){:}$

11 $$\begin{aligned} S_i(t) - a(t-t_i)< S_k(t) \Rightarrow m \cdot \left( \frac{r^{-\alpha } - (r+1)^{-\alpha }}{\sum _{j=1}^N j^{-\alpha }}\right) < a(t-t_i). \end{aligned}$$

The above describes the maximum age for one topic to stay on rank *r* with the given $m, a, \alpha$ and *N*, leading directly to a lower bound of the average time $\tau$ spent in a rank *r*:

12 $$\begin{aligned} \text {min}\left(\langle \tau (r) \rangle \right) \sim r^{-\alpha } - (r+1)^{-\alpha }. \end{aligned}$$

**Fig. 13:**
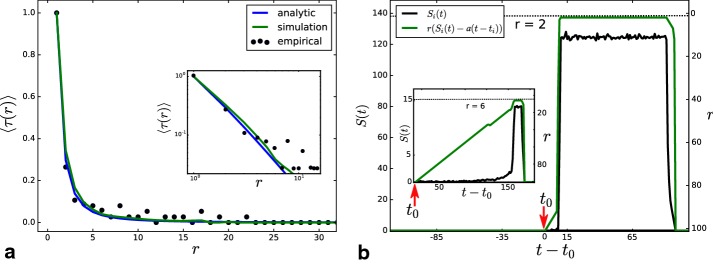
Average times of stable ranks and exemplary trajectories: **a** the analytic lower bound from Eq. (12), the resulting times from the Monte-Carlo simulation and the empirical values of the normalized average time $\langle \tau (r) \rangle$ of stable ranking *r*. The values are normalized to their maximum at $r=1$ to make them comparable. The inset shows the same plot with logarithmic axes. **b** Two trajectories from the simulation that reach different maximal ranks. The size $S_i(t)$ in black and the rank $r(S_i(t)-(t-t_i))$ in green, which reaches a maximum of $r=2.$ The inset shows the same quantities for an item that reaches only $r=6$

The above can be compared with the resulting average times $\langle \tau (r) \rangle$ of topics staying in one rank in the simulation and the empirical dataset. Figure 13a shows the results, where it becomes obvious that higher-ranked hashtag groups can keep their ranking longer than the smaller ones. The empirical data (dots) confirm that relation very well, especially for high ranks. One can observe in the inset (log–log plot) that for lower ranks (ca. $r > 6$), the approximation of the perfect opponent overestimates the competition in the lower ranks where not only the fittest can overtake. An example trajectory in Fig. 13b shows that, for items that reach high ranks, the approximation of an adiabatic rise holds since it reaches its maximum at a young age. In the inset, an item that only reaches rank 6 is shown. This happens quite late in the lifetime after a gradual climb up, so the assumption of young competitors does not hold here.

## Conclusion

We have presented an investigation to analyze the dynamic behavior of topics in online media. We have focused on a dataset of hashtags, which were used on the fashion platform: lookbook.nu over the course of 1 year.

We have built timestamped co-occurrence networks and aggregated them to weighted snapshot graphs. We have applied a random-walk-based approach for finding a transition and a community region to obtain a reliable and meaningful clustering.

Independent from the method for community detection, we have proposed a construction of weighted bipartite networks of successive timesteps to track group dynamics over time. For robustness against temporal fluctuations and instabilities, we have extended the Jaccard index, determining the weights, to incorporate higher-order memory.

The resulting dynamics show fat-tailed distributions of relative gains and losses, characterizing bursty behaviors in the increases and also decreases of hashtag groups. In order to describe and understand these developments, we have formulated a ranking model that incorporates gain and loss from a combined attractiveness score of community size and age.

Based on the model results and affirmed by the empirical findings, competition among ranked items with unlasting prestige scores can lead to bursty behaviors in the gains and also losses of popularity. In addition, we found that competition becomes intense for higher ranks so they have to be reached in a young age. The simple model can be further extended and is applicable to other online media where recency plays an important role.
